# Difficult Management of a Double-Lumen Endotracheal Tube and Difficult Ventilation during Robotic Thymectomy with Carbon Dioxide Insufflation

**DOI:** 10.1155/2017/3403045

**Published:** 2017-04-26

**Authors:** Yuki Sugiyama, Kunihiro Mitsuzawa, Yuki Yoshiyama, Fumiko Shimizu, Satoshi Fuseya, Takashi Ichino, Hiroyuki Agatsuma, Takayuki Shiina, Ken-ichi Ito, Mikito Kawamata

**Affiliations:** ^1^Department of Anesthesiology and Resuscitology, Shinshu University School of Medicine, Matsumoto, Japan; ^2^Division of Breast, Endocrine and Respiratory Surgery, Department of Surgery (II), Shinshu University School of Medicine, Matsumoto, Japan

## Abstract

Robotic surgery with carbon dioxide (CO_2_) insufflation to the thorax is frequently performed to gain a better operative field of view, although its intraoperative complications have not yet been discussed in detail. We treated two patients with difficult ventilation caused by distal migration of a double-lumen endotracheal tube (DLT) during robotic thymectomy. In the first case, migration of the DLT during one-lung ventilation (OLV) occurred after CO_2_ insufflation to the bilateral thoraxes was started. Oxygenation rapidly deteriorated because dependent lung expansion was restricted by CO_2_ insufflation. In the second case, migration of the DLT during OLV occurred while CO_2_ insufflation to a unilateral thorax and mediastinum was performed. In both cases, once migration of the DLT during OLV occurred with CO_2_ insufflation, readjusting the DLT became very difficult because our manipulation of bronchofiberscopy was prevented by the robot arms located above the patient's head and because deformation of the trachea/bronchus induced by CO_2_ insufflation caused a poor image of the bronchofiberscopic view. Thus, during robotic-assisted thoracoscopic surgery with CO_2_ insufflation, since there is a potential risk of difficult ventilation with a DLT and since readjustment of the DLT is very difficult, discontinuing CO_2_ insufflation and switching to double-lung ventilation are needed in such a situation.

## 1. Introduction

Robotic-assisted thoracoscopic surgeries have recently provided a technical advance for overcoming the many limitations of conventional thoracoscopic surgeries and have gained widespread popularity in a clinical setting [1]. Carbon dioxide (CO_2_) insufflation to a unilateral thorax or bilateral thoraxes is frequently performed to gain a better operative field of view, although its intraoperative complications have not yet been discussed in detail. Here we report two cases of sudden onset of difficult ventilation due to a migrated double-lumen endotracheal tube (DLT) during robotic thymectomy. We highlight the difficulty in readjustment of the migrated DLT during robotic thymectomy with continuing CO_2_ insufflation.

## 2. Case Presentation

### 2.1. Case  1

A 40-year-old woman (height, 160 cm; weight, 60 kg) with ocular myasthenia gravis was scheduled for robotic thymectomy. Computed tomography (CT) revealed a thymoma of 35 mm in diameter in the anterior mediastinum. Her trachea was not deviated and her left mainstem bronchus was 40 mm in length. Preoperative pulmonary function values were normal. Arterial oxygen tension (PaO_2_) and carbon dioxide tension (PaCO_2_) under room air were 70.4 mmHg and 45.1 mmHg, respectively. The rest of the preoperative examination was unremarkable.

General anesthesia was induced and the trachea was intubated with a 35-French left-sided DLT (Broncho-Cath®, COVIDIEN, Dublin, Ireland) under bronchofiberscopic guidance, confirming that the proximal bronchial cuff end was located about 5 mm distal from the carina. The DLT was fixed at a depth of 27 cm at the right angle of the mouth. Surgery was initiated in the supine position, and left one-lung ventilation (OLV) was started. CO_2_ insufflation to the right thorax was started at 5 mmHg and then increased to 10 mmHg. She was ventilated by pressure-controlled ventilation, volume-guaranteed mode (PCV-VG®, GE Healthcare, Little Chalfont, England) at a tidal volume (TV) of 450 mL, respiratory rate of 12 breaths/min, positive end-expiratory pressure (PEEP) of 5 cmH_2_O, and upper limit of peak inspiratory pressure (PIP) of 30 cmH_2_O, with 50% oxygen. The robot arms (da Vinci Surgical System SI®, Intuitive Surgical Inc., Sunnyvale, CA, USA) were then connected to the thoracic port, and robotic thymectomy was performed.

**Figure 1:**
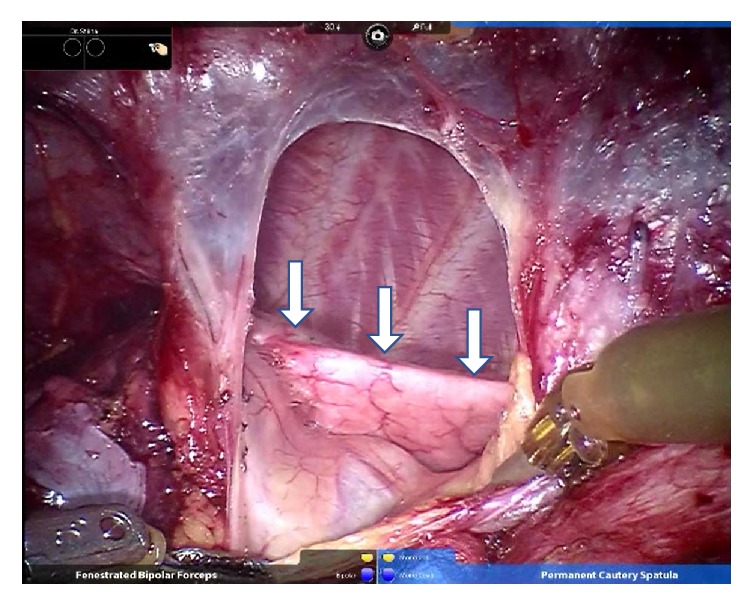
Restriction of left (dependent) lung expansion including the upper lobe of the left lung (arrow) during the inspiratory phase after an incision of the left mediastinal pleura.

**Figure 2:**
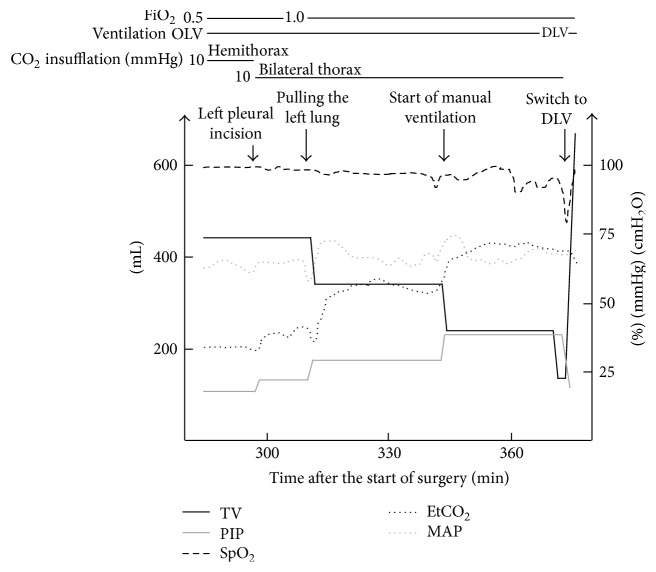
Changes of vital signs and ventilator settings in case  1. TV, tidal volume; PIP, peak inspiratory pressure; EtCO_2_, end-tidal carbon dioxide; MAP, mean arterial pressure; OLV, one-lung ventilation; DLV, double-lung ventilation.

Three hours after starting surgery, the left mediastinal pleura was incised to divide adhesions between the tumor and the left lung, and CO_2_ was insufflated to the bilateral thoraxes at 10 mmHg. In the operative field, the left (dependent) lung contracted rapidly and its expansion was severely interrupted (Figure 1). Although SpO_2_ was maintained at 98% with 50% oxygen, end-tidal CO_2_ (EtCO_2_) and PIP increased from 38 to 40 mmHg and 20 to 23 cmH_2_O, respectively (Figure 2). Ten minutes after left pleural incision, TV decreased to 350 mL and PIP increased to 30 cmH_2_O of the upper limit while the surgeon was pulling the left lung and dividing the adhesion between the tumor and the left lung. We attempted to assess the DLT position by bronchofiberscopy from the tracheal lumen; however, the space between the end of the tracheal lumen and tracheal wall was so narrow that we could not find the carina and bronchial cuff. The patient's cart and robot arms of da Vinci located just above her head prevented the manipulation of bronchofiberscopy. Inspired oxygen was increased to 100% and manual ventilation was started. TV reached only 250 mL at 40 cmH_2_O PIP and EtCO_2_ increased to 60 mmHg. SpO_2_ simultaneously decreased to 90%. Bronchofiberscopy from the bronchial lumen was avoided because hypoxia had already occurred and interrupting ventilation was unavailable. The surgeon pointed out that only the left lower lobe was ventilated, although we could not find where the bronchial cuff was located by bronchofiberscopy. Finally, TV decreased to 150 mL at 40 cmH_2_O PIP, and SpO_2_ decreased to 80%.

At that point, CO_2_ insufflation was discontinued, and the robot was promptly disconnected. We immediately started double-lung ventilation. PIP decreased to 20 cmH_2_O and TV increased to 700 mL. The bronchofiberscopic view was improved and we found that the bronchial cuff was distally located. When we tried to readjust the position, the DLT was accidentally extubated, and then a single-lumen tube was used for intubation. We inserted a bronchial blocker (COOPDECH® endobronchial blocker, Daiken-iki, Osaka, Japan), and left OLV was resumed. The operation was restarted in thoracoscopic surgery and completed thereafter uneventfully without conversion. Operative time was 327 minutes and blood loss was less than 100 mL. She was extubated in the operating room and transferred to an intensive care unit (ICU). She was discharged from the ICU on postoperative day 1 and was discharged from the hospital on postoperative day 8 without complications.

### 2.2. Case  2

A 51-year-old man (height, 169 cm; weight, 109 kg) without symptoms was scheduled for robotic thymectomy. CT revealed a thymoma of 27 mm in diameter in the anterior mediastinum. His trachea was not deviated and his left mainstem bronchus was 50 mm in length. Preoperative pulmonary function values were normal. PaO_2_ and PaCO_2_ under room air were 73.2 mmHg and 39.1 mmHg, respectively. He was being treated for hypertension and sleep apnea syndrome. The rest of the preoperative examination was unremarkable.

General anesthesia was induced, and the trachea was intubated with a 37-French left-sided DLT under bronchofiberscopic guidance, confirming that the proximal bronchial cuff end was located about 3 mm distal from the carina. The DLT was fixed at a depth of 29 cm at the left angle of the mouth. Surgery was initiated in a low Fowler's position. Left OLV was started and CO_2_ insufflation to the right thorax was started at 5 mmHg and then increased to 10 mmHg. He was ventilated by pressure-controlled ventilation at PIP of 30 cmH_2_O, respiratory rate of 12 breaths/min, and PEEP of 10 cmH_2_O, with 100% oxygen. The robot arms were then connected to the thoracic port, and robotic thymectomy was performed. The DLT position at 20 minutes after starting surgery was the same as that at the time of intubation.

**Figure 3:**
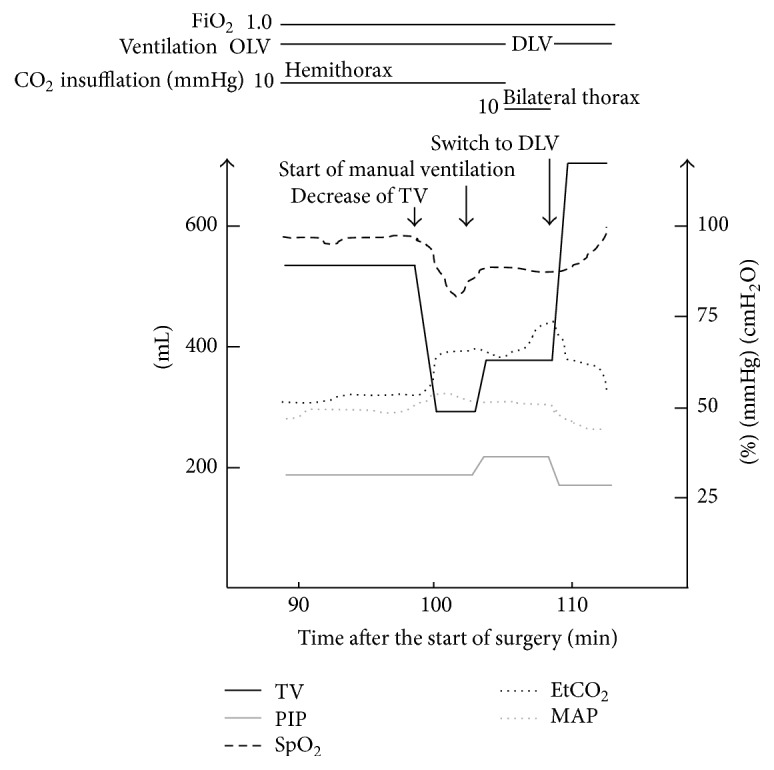
Changes of vital signs and ventilator settings in case  2. TV, tidal volume; PIP, peak inspiratory pressure; EtCO_2_, end-tidal carbon dioxide; MAP, mean arterial pressure; OLV, one-lung ventilation; DLV, double-lung ventilation.

One hundred minutes after starting surgery, TV suddenly decreased from 550 mL to 160 mL, and SpO_2_ was decreased to 82% (Figure 3). Manual ventilation was started at PIP of 35 cmH_2_O. TV reached 300 mL and SpO_2_ was increased to 88%. We attempted to assess the DLT position by bronchofiberscopy; however, as in case  1, the bronchofiberscopic view was extremely poor and the robot arms prevented manual manipulation. The DLT seemed to be distally placed, but this was not convincing. Although surgeons incised the left mediastinal pleura, the situation was unchanged and they did not find a nonventilated lobe.

At that point, CO_2_ insufflation was discontinued and then double-lung ventilation was immediately started. PIP decreased to 28 cmH_2_O and TV increased to 730 mL. The bronchofiberscopic view was improved and distal migration of the DLT was confirmed. The DLT was pulled out about 8 mm for readjustment, and then left OLV with CO_2_ insufflation at 5 mmHg was resumed. Thereafter, the robotic surgery restarted and completed uneventfully without conversion. Operative time was 281 minutes and blood loss was 300 mL. He was extubated in the operating room and discharged from the hospital on postoperative day 6 without complications.

## 3. Discussion

The introduction of robotic surgery is the most significant advance in minimally invasive surgery of this decade. Robotic-assisted thoracoscopic surgery has been tested in various thoracic surgery procedures including thymectomy [2–5]. The three-dimensional vision system and articulating instruments of the da Vinci Surgical Robotic System enable an intuitive “open-like” intervention with minimally invasive access [6, 7]. CO_2_ insufflation to the thorax has been used for robotic-assisted thoracoscopic surgery to gain a better operative field of view by requests from surgeons. In robotic thymectomy, the mediastinal pleurae on both sides are intentionally incised in order to confirm the phrenic nerve location [8–12]; therefore, CO_2_ is sometimes insufflated to the bilateral thoraxes. The pressure of CO_2_ insufflation to the thorax is usually maintained at 5–15 mmHg [9–12], although caution has been raised about difficult ventilation [11, 12].

In our cases, distal migration of the DLT occurred during CO_2_ insufflation to the thorax while the surgeon was manipulating the lesion site. Although migration of a DLT due to the surgical procedure is not rare in anesthetic management of OLV, we should know that CO_2_ insufflation itself also has a potential risk of DLT migration. It has been reported that hypoxia due to difficult ventilation caused by distal migration of a DLT occurred just after the start of CO_2_ insufflation during robotic thymectomy and that the migration of the DLT was thought to be caused by mediastinal shifting induced by CO_2_ insufflation [12]. Similarly, in our cases, distal migration of the DLT was thought to be caused not only by the surgical procedure but also by CO_2_ insufflation. Conceivably, a DLT during CO_2_ insufflation would easily migrate distally as was seen in our cases and in a previous case report [12]. We placed the DLT at a relatively shallow position in case  2, though difficult ventilation due to distal migration of the DLT occurred. Thus, we need to pay attention to appropriate placement of the bronchial tip of the DLT not only before and after starting of CO_2_ insufflation but also throughout CO_2_ insufflation. Another possible cause of migration of a DLT is the position of the patient. Although it is unclear whether distal migration of a DLT occurs more easily in the supine positon or the lateral decubitus positon during robotic-assisted thoracoscopic surgery, the lateral decubitus position itself often causes migration of a DLT [13]. Thus, reconfirming placement of the tip of the DLT after postural change is indispensable.

CO_2_ insufflation to the bilateral thoraxes per se has the potential risk of hypoxia because of forced restriction of dependent lung expansion due to partial collapse of the distal trachea and bronchus. Regarding the impact of CO_2_ insufflation on respiratory factors, a previous study on robotic coronary artery bypass grafting showed that CO_2_ insufflation to bilateral thoraxes increased PIP by 5 mmHg and PaCO_2_ by 15 mmHg [14]. In case  1, increases in PIP and EtCO_2_ were observed at the time of insufflation to bilateral thoraxes, suggesting that thoracoscopic surgery without CO_2_ insufflation or open surgery should be considered in patients with respiratory complications. We also need to keep in mind that these risks of CO_2_ insufflation to the thorax are not only for robotic surgery but also for all thoracoscopic surgeries.

The restricted space caused by the robot system is another specific problem for anesthesiologists during robotic-assisted thoracoscopic surgery. In the simulation using da Vinci, we realized that the patient cart and robot arms of da Vinci located above the patient's head were obstacles for anesthesiologists and we adjusted the position to keep as much space as possible, although the space was not enough during the actual surgery. A new robotic system such as da Vinci Xi, which has thin arms and instruments with a long reach, may solve this problem.

Thus, once difficult ventilation occurs during OLV with CO_2_ insufflation in the process of robotic-assisted thoracoscopic surgery, readjusting of the DLT is very difficult and hypoxia occurs immediately; therefore, if difficult ventilation and/or hypoxia occur, CO_2_ insufflation should be discontinued and double-lung ventilation should be started as soon as possible. After stabilization of vital signs and improvement of ventilation, assessment and readjustment of the DLT position should be tried. It should be easy to confirm the position of the DLT after discontinuation of CO_2_ insufflation because the space between the DLT and tracheal/bronchial wall would be expanded and the poor image of the bronchofiberscopic view due to deformation of the trachea/bronchus induced by CO_2_ insufflation would be improved as was seen in our two cases. Using a single-lumen tube with a bronchial blocker is another choice for robotic-assisted thoracoscopic surgery. This technique has already been applied to robotic mediastinal surgeries [8, 15].

In conclusion, we experienced two cases of difficult readjustment of a migrated DLT that resulted in difficult ventilation during robotic thymectomy. It is indispensable for surgeons and anesthesiologists to achieve a consensus that if difficult ventilation occurs, discontinuation of CO_2_ insufflation and restarting double-lung ventilation should be done as soon as possible.
